# Differential Vulnerability of Oculomotor Versus Hypoglossal Nucleus During ALS: Involvement of PACAP

**DOI:** 10.3389/fnins.2020.00805

**Published:** 2020-08-11

**Authors:** Grazia Maugeri, Agata Grazia D’Amico, Giovanna Morello, Dora Reglodi, Sebastiano Cavallaro, Velia D’Agata

**Affiliations:** ^1^Department of Biomedical and Biotechnological Sciences, Section of Anatomy, Histology and Movement Sciences, University of Catania, Catania, Italy; ^2^Department of Drug Science, University of Catania, Catania, Italy; ^3^Institute for Biomedical Research and Innovation (IRIB), National Research Council (CNR), Catania, Italy; ^4^Department of Anatomy, MTA-PTE PACAP Research Team, University of Pécs Medical School, Pécs, Hungary

**Keywords:** amyotrophic lateral sclerosis, lower motor neurons, oculomotor nucleus, hypoglossal nucleus, pituitary adenylate cyclase-activating polypeptide

## Abstract

Amyotrophic lateral sclerosis (ALS) is a progressive multifactorial disease characterized by the loss of motor neurons (MNs). Not all MNs undergo degeneration: neurons of the oculomotor nucleus, which regulate eye movements, are less vulnerable compared to hypoglossal nucleus MNs. Several molecular studies have been performed to understand the different vulnerability of these MNs. By analyzing postmortem samples from ALS patients to other unrelated decedents, the differential genomic pattern between the two nuclei has been profiled. Among identified genes, adenylate cyclase activating polypeptide 1 (ADCYAP1) gene, encoding for pituitary adenylate cyclase-activating polypeptide (PACAP), was found significantly up-regulated in the oculomotor versus hypoglossal nucleus suggesting that it could play a trophic effect on MNs in ALS. In the present review, some aspects regarding the different vulnerability of oculomotor and hypoglossal nucleus to degeneration will be summarized. The distribution and potential role of PACAP on these MNs as studied largely in an animal model of ALS compared to controls, will be discussed.

## Introduction

Amyotrophic lateral sclerosis (ALS) is an incurable and multifactorial neurodegenerative disease induced by the synergistic action of genetic and environmental factors. The ALS cases are classified into sporadic (SALS) and familial (FALS) forms. Mutations in the Cu/Zn superoxide dismutase 1 (SOD1) gene account for approximately 20% of FALS cases (Rosen et al., 1993). For the most of ALS patients, disease etiology is unknown and pathological events leading to degeneration of upper and lower motor neurons (MNs) start long before the appearance of clinical symptoms. Not all MNs undergo degeneration during the progression of ALS. In fact, somatic MNs of the oculomotor nucleus controlling eye movements are generally spared compared to the more vulnerable hypoglossal MNs innervating the tongue (Mannen et al., 1977; Reiner et al., 1995; Nimchinsky et al., 2000; Haenggeli and Kato, 2002; Hedlund et al., 2010). It has been suggested that phenotypic and genetic heterogeneity of MNs is responsible for the different susceptibility to injury (Hedlund et al., 2010).

Through microarray technology, Hedlund et al. (2010) compared the genomic pattern of oculomotor to hypoglossal nucleus. Among identified genes, adenylate cyclase activating polypeptide 1 (ADCYAP1) gene, encoding for pituitary adenylate cyclase-activating polypeptide (PACAP), is significantly up-regulated in the oculomotor as compared to hypoglossal nucleus suggesting a possible role of the peptide in the higher resistance of oculomotor MNs to death.

The involvement of PACAP in ALS has been described in several papers. Comparison of microarray datasets of sporadic ALS motor cortex and mutated SOD1 (mSOD1) G93A mice brains revealed that some genes, including those of PACAP and its receptor PAC1R, are deregulated in the same direction in both human and transgenic animals (Morello et al., 2017b).

The cellular and regional distribution of PACAP and its receptors, known as PAC1R, VPAC1, and VPAC2 receptors, has been extensively investigated in central and peripheral nervous system. In response to neurodegenerative insult, the peptide is released from MNs and exerts a neuroprotective role through autocrine and/or paracrine mechanisms mediated by cAMP/PKA-(cyclic adenosine monophosphate/protein kinase A) or PI3K (phosphoinositol three kinase) pathways (Manecka et al., 2013). Moreover, it has also been demonstrated that the trophic effect of PACAP can be exerted either directly or indirectly through EGFR trans-activation.

The present review aims to summarize findings on the role played by PACAP in the neurodegenerative process affecting lower MNs during ALS on postmortem samples of decedents with ALS and in an animal model of ALS, mainly focusing on the oculomotor and hypoglossal nuclei.

## Overview on ALS and Different Vulnerability of Oculomotor Versus Hypoglossal Nucleus MNS to Degeneration

ALS is a motor neuron disease, a degenerative, progressive and paralytic disorder targeting MNs of the brain and spinal cord (Rowland and Shneider, 2001). The early stage of the disease is characterized by focal weakness progressing toward the impairment of most muscles including the diaphragm (Wijesekera and Leigh, 2009). Patients’ mean survival from onset is ∼3 years and the most common cause of death is respiratory paralysis. In United States and Europe, the incidence of ALS is one case per 100,000 people per year. Its prevalence drastically increases with age (Johnston et al., 2006; Chiò et al., 2013; Robberecht and Philips, 2013).

**FIGURE 1:**
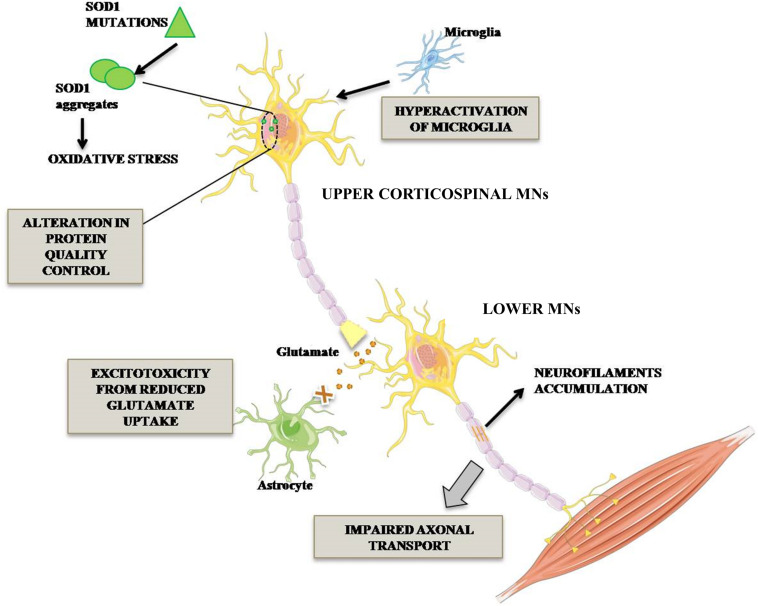
Pathogenetic mechanisms occurring in mutant SOD1 MNs. ALS-associated mutation in Cu/Zn SOD1 triggers complex events leading to MN degeneration. In particular, impairment of axonal transport of mitochondria in MNs, alteration in protein degradation and aberrant activation of microglia triggering neuroinflammatory process. Lower MN neurodegeneration is also promoted by glutamate-mediated excitotoxicity.

The ALS cases are classified in sporadic (SALS) and familial (FALS) forms. The first one, without genetic inherited component, occurs in 90% of cases, while FALS cases (10%) are associated with mutations identified in distinctive genes. Among these, superoxide dismutase 1 (SOD1) was the first mutated gene to be discovered about two decades ago and it accounts for 20% of FALS cases (Rosen et al., 1993). More than 160 mutations in SOD1 gene, including G93A, A4V, H46R, and D90A have been reported. The toxic mechanism through which SOD1 mutations lead to MNs degeneration is not completely clarified. However, it is known that SOD1 mutant protein is overexpressed, misfolded and elicits various toxic effects such as increased oxidative stress, activation of microglia leading to inflammation, alteration of protein quality control due to proteasome defect, excitotoxicity due to decreased glutamate re-uptake and alteration of axonal transport (Figure 1; Bristol and Rothstein, 1996; Hoffman et al., 1996; Grosskreutz et al., 2010).

During the last 15 years, other FALS mutated genes have been identified, including TAR DNA binding protein (TARDBP), Fused in Sarcoma (FUS) and Chromosome 9 open reading frame 72 genes (C9orf72). TDP43, encoded by the most common mutated ALS gene TARDBP, and FUS proteins regulate RNAs expression and maturation (Neumann et al., 2006; Lagier-Tourenne et al., 2010; DeJesus-Hernandez et al., 2011). Additional ALS risk genes comprise: heterogenous nuclear ribonucleoprotein A1 (HNRNPA1) and heterogenous nuclear ribonucleoprotein A2B1 (HNRNPA2B1) both involved in pre-mRNA processing, metabolism and transport; profilin 1 (PFN1) binding to actin and affecting cytoskeleton structures; ataxin-2 (ATXN2) modulating RNA processing; chromatin modifying protein 2B (CHMP2B) involved in recycling and degradation of cell surface receptors; ubiquilin 2 (UBQLN2) regulating protein degradation; vesicle-associated membrane associated-protein B (VAPB) and the ATP-binding protein valosin-containing protein (VCP) both involved in membrane and vesicle trafficking; optineurin (OPTN), TANK-Binding Kinase 1 (TBK1) and sequestosome 1 (SQSTM1), which are implicated in normal protein autophagy (Ajroud-Driss and Siddique, 2015). Although the above mentioned FALS genes are expressed in multiple cell types, their mutation induces a selective degeneration of MNs.

**FIGURE 2:**
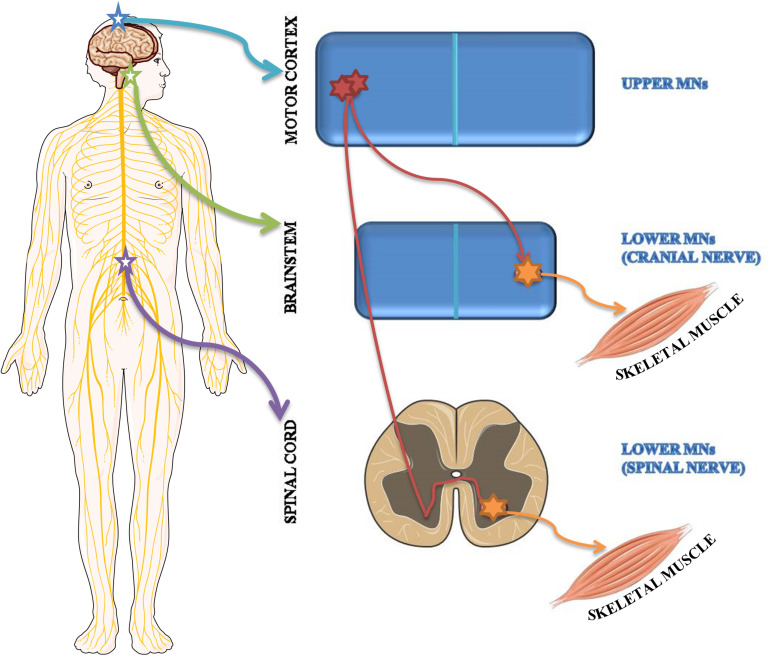
Schematic representation of upper and lower MN localization. Upper MNs originate in the motor region of the cerebral cortex. Lower MNs are located either in some cranial nerve nuclei of the brainstem as well as in the ventral horns of the spinal cord.

Based on brain localization, MNs are classified as upper MNs located in the motor cortex and lower MNs situated in brainstem motor nuclei and in spinal cord ventral horns (Figure 2).

Although upper and lower MNs are both affected during the disease, lower MNs show a different susceptibility to degeneration. From disease onset, MNs localized in ventral horns of spinal cord and hypoglossal nucleus begin to degenerate, whereas MNs of oculomotor, trochlear and abducens nuclei controlling eye movements are spared (Mannen et al., 1977; Reiner et al., 1995; Nimchinsky et al., 2000; Haenggeli and Kato, 2002; Hedlund et al., 2010). In accord, during disease progression patients unable to speak continue to communicate through eye-tracking devices requiring the use of eye movements.

Typical clinical symptoms linked to upper MN degeneration comprise spasticity, uncontrolled movement and significant reduction of sensitivity (Ivanhoe and Reistetter, 2004). Lower MNs receive inputs from the upper population, sensory neurons and interneurons. Their degeneration induces loss of synaptic connectivity leading to muscular atrophy and, at the end, paralysis (Stifani, 2014).

In the present paper, we are focusing on data regarding the different vulnerability of lower MNs in oculomotor versus hypoglossal nucleus. The mechanism underlying their different death susceptibility is not fully understood, but it is clear that the resistance of some MNs to degeneration is related to anatomical specificity and their transcriptome profile.

**FIGURE 3:**
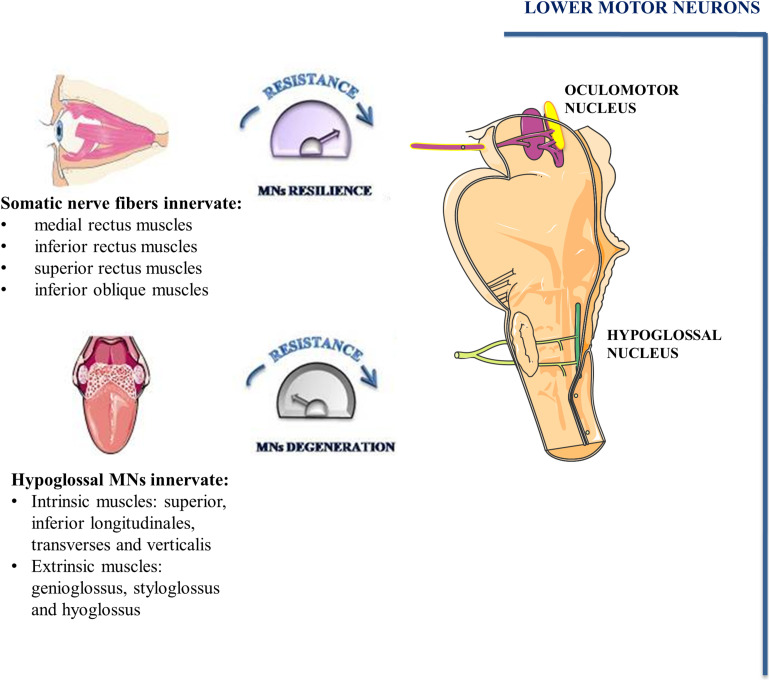
Oculomotor and hypoglossal nerve. Somato-motor fibers of oculomotor nucleus (pink) as well as autonomic fibers of accessory parasympathetic nucleus (yellow) form the oculomotor nerve. Somato-motor fibers innervate EOMs involved in eye movements. MNs of hypoglossal nuclei innervate the muscles of the tongue and undergo degeneration during the early phase of ALS.

The oculomotor nucleus, adjoining to trochlear nucleus, is situated at the level of the superior colliculus in the midbrain and it extends rostrally up to the posterior commissure. By examining its ultrastructure in rat brains, it has been demonstrated that it comprises either large MNs with abundant cytoplasm and well-developed organelles as well as small MNs with a low amount of cytoplasm. Although axo-somatic and axo-dendritic synapses are the most prevalent, axo-axonic synapses have also been identified. Fibers of the oculomotor nucleus as well as autonomic fibers of the accessory parasympathetic nucleus (Edinger-Westphal nucleus) converge into the oculomotor nerve (cranial nerve-CNIII). Somatic nerve fibers are bundled inside the nerve and are surrounded by the autonomic ones (Figure 3; Büttner-Ennever, 2006).

In mammals, conjugate eye movement results from combined contraction of six striated muscles inserted on the external surface of the eye bulb. Four of the six extraocular muscles (EOMs), known as medial rectus, inferior rectus, superior rectus and inferior oblique muscles are innervated by MNs of oculomotor nucleus. The superior oblique muscle is innervated by trochlear nerve MNs whereas the abducens nucleus MNs innervate the lateral rectus muscle.

EOMs comprise six different types of fibers, including those with very high mitochondrial content and strong fatigue resistance. They show a more complex pattern of innervation than almost all skeletal muscles. In particular, they have neuromuscular junctions extensively dispersed along their length from origin to muscle insertion (Harrison et al., 2007). Furthermore, they are characterized by ten distinct types of myosin heavy chain fibers within a single myofibre (Zhou et al., 2011). Moreover, EOMs contain several negative regulators of complement system showing higher vulnerability to myasthenia gravis, a neurotransmission disorder (Spencer and Porter, 1988). In EOMs, MNs form motor units of small size (i.e., MNs/muscles fibers ratio 1:300 for EOMs; 1:2000 for large skeletal muscle), moreover, a single MN axon innervates the same muscles fibers in different regions allowing a fine regulation of EOMs contraction.

Hypoglossal nucleus is localized on the floor of the fourth ventricle in the dorsal part of the medulla oblongata. It contains a high number of MNs and inhibitory interneurons. MNs of the hypoglossal nucleus show large soma containing high levels of Nissl substance (Boone and Aldes, 1984). Their axons emerge from the preolivary fissure forming the hypoglossal nerve (CN XII). All tongue intrinsic and extrinsic muscles are innervated by XII nerve (Figure 3). The tongue intrinsic muscles, including superior, inferior longitudinales, transversus and verticalis muscles allow to change the organ shape. Among extrinsic muscles, genioglossus draws the tongue forward from the root, stíloglossus upward, whereas hyoglossus exerts retraction of the tongue and its side depression. The contraction of these muscles is involved in vital functions such as mastication, swallowing, suckling, vocalization and respiration.

In addition to the anatomical and functional differences, oculomotor and hypoglossal nuclei MNs have also a different genes expression pattern. By using laser capture microdissection and microarray analysis in a SOD1^G93A^ rat model of ALS, Hedlund et al. (2010) showed profound differences in gene expression patterns when motor neurons of the oculomotor/trochlear complex (which do not degenerate in ALS) were compared to motor neurons of hypoglossal and the lateral motor column of the cervical enlargement of the spinal cord (which show vulnerability in ALS).

In a previous paper, Aronica et al. (2015), have analyzed whole-genome expression profile of motor cortex in sporadic ALS (SALS) and healthy patients. Unsupervised hierarchical clustering analysis allowed to discriminate controls from SALS patients, classified in SALS1 and SALS2 subtypes, each linked to differentially expressed genes and biological pathways (Morello et al., 2017a). Particularly, the most representative functional processes deregulated in SALS1 were involved in the regulation of chemotaxis, immune response, cell adhesion, signal transduction and communication. Deregulated genes in SALS2, in turn, were selectively associated with cell adhesion and cytoskeleton organization, regulation of transport and mitochondrial oxidative phosphorylation, energy metabolism and apoptotic signaling cascade (Aronica et al., 2015).

By comparing gene expression profile data, we have found that majority of genes deregulated in oculomotor/hypoglossal nuclei (127/188; Supplementary Table) are also deregulated in motor cortex of SALS1 and SALS2 patients (Aronica et al., 2015). Moreover, some of these genes, such as Igf2, and Gda, encode products exerting neuroprotection from glutamate-induced toxicity (Hedlund et al., 2010). The data suggest that the unique genome profile and specific protein signature could in part explain the differential vulnerability of these nuclei in ALS. Interestingly, overexpression of PACAP encoded by the ADCYAP1 in the in the oculomotor nucleus (Hedlund et al., 2010) should result in a trophic effect as demonstrated in ALS and other neurodegenerative models (Morio et al., 1996; Shintani et al., 2005; Wu et al., 2006).

## PACAP and ALS

PACAP is a neuropeptide identified for the first time in the ovine hypothalamus and belonging to VIP/secretin/glucagon family (Arimura, 1998). There are two functional isoforms: PACAP38 with 38 amino acids residues and PACAP27 including the N-terminal 27 amino acids residues of PACAP38 (Sherwood et al., 2000). It exerts different functions through activation of three distinct G-protein-coupled receptors: PAC1R, VPAC1R, and VPAC2R, whose expression is tissue and cell type-specific (Harmar et al., 2012). These receptors activate different signaling cascades including protein kinase A (PKA), protein kinase C (PKC) (Vaudry et al., 2009), MAPKs (mitogen-activated protein kinases) (Lelièvre et al., 1998) and NF-kB signaling pathways (Delgado and Ganea, 1999).

PACAP and its receptors are expressed in different tissues and organs, where they are involved in several biological processes including cell division and survival (Canonico et al., 1996; D’Agata et al., 1996; Jaworski, 2000; Maugeri et al., 2018). PACAP is also involved in learning and memory (Sacchetti et al., 2001; Adamik and Telegdy, 2005; Ciranna and Costa, 2019) and plays protective role in different neurodegenerative diseases (Reglodi et al., 2004, 2011; Waschek, 2013).

Tissue distribution studies have demonstrated that PACAP mRNA precursor is present in cerebral cortex, olfactory bulb, cingulate cortex, dentate gyrus, CA1 and CA4 subregions of the hippocampus and cerebellum in the developing and adult rat brain (Arimura et al., 1991; Ghatei et al., 1993). PACAP-immunoreactivity was found in hypothalamic and extrahypothalamic brain sites including the preoptic area, dorsomedial and arcuate hypothalamic nuclei, pontine parabrachial nucleus, nucleus of the solitary tract and dorsal motor vagal nucleus rat brain (Légrádi et al., 1994; Hannibal, 2002). PACAP positive signal was also detected in the superficial layer of the superior colliculus, in the midline between the periaqueductal gray and the ventral tegmental area including the two oculomotor nuclei (Shioda et al., 1997). Low PACAP density immunostaining was found in the fibers and cell bodies of hypoglossal nucleus MNs (Légrádi et al., 1994).

A review paper has highlighted that PAC1R has greater expression than VPAC1-R and VPAC2-R receptors in the CNS (Basille et al., 2000). In particular, PAC1R is highly expressed in the olfactory bulb, the dentate gyrus, the supraoptic nucleus of the hypothalamus, the cerebellar cortex and hypoglossal nucleus whereas it is weakly expressed in oculomotor nucleus (Shioda et al., 1997; Skoglosa et al., 1999).

The analysis of genome expression profile ALS patients motor cortex has highlighted a significant deregulation either in PACAP and PAC1R mRNAs. In particular, PACAP is over-expressed in SALS1 and down-regulated in SALS2 motor-cortex samples, whereas PAC1R is down-regulated in both subgroups as compared to controls. To evaluate the role of PACAP in MNs degeneration, the effect of peptide has been tested in an *in vitro* model of MNs-derived from human induced pluripotent stem cells (iPSC) exposed to neurodegenerative insult (Bonaventura et al., 2018). The results have shown that PACAP and PAC1R levels are up-regulated in MNs cultured in growth factors deprived medium. Moreover, exogenous PACAP treatment effectively prevented their apoptotic death (Bonaventura et al., 2018). In accord, previous papers have suggested that endogenous PACAP promotes MNs survival following exposure to different insult through an autocrine or paracrine mechanism (Ohsawa et al., 2002; Armstrong et al., 2003; Chen and Tzeng, 2005; Tomimatsu and Arakawa, 2008; Bonaventura et al., 2018). Particularly, in response to adult rat facial nerve axotomy, a robust time-dependent increase in PACAP as well as decrease in PAC1R mRNA was observed in the facial motor nucleus as compared to the contralateral side (Zhou et al., 1999; Waschek et al., 2000). The endogenous PACAP released from the distal nerve stump binds to PAC1R expressed on Schwann cells during regeneration (Woodley et al., 2019). After nerve injury, PACAP was up-regulated and detectable in facial motor nucleus until 1 month, corresponding to the period of axon regeneration (Reimer et al., 1999). Moreover, PACAP deficiency in PACAP knock-out mice leads to a significant delay of axonal regeneration of axotomized facial nerve confirming the role of PACAP in axonal regeneration process (Armstrong et al., 2008). On the other hand, it is possible to speculate that PAC1R downregulation after nerve axotomy may correlate to increased apoptotic death of MNs of facial motor nucleus (Mattsson et al., 2006).

Therefore, it is possible to speculate that over-expression of PACAP encoding gene, ADCYAP1, in oculomotor nucleus may be implicated in its less vulnerability during ALS degeneration. In accord to this hypothesis, PACAP was found up-regulated in the axotomized facial motor nuclei of transgenic SOD1 G93A mice (Mesnard et al., 2011). Furthermore, Chung et al. (2005) demonstrated that higher resistance of ventral tegmentum compared to substantia nigra is partially due to PACAP expression.

**FIGURE 4:**
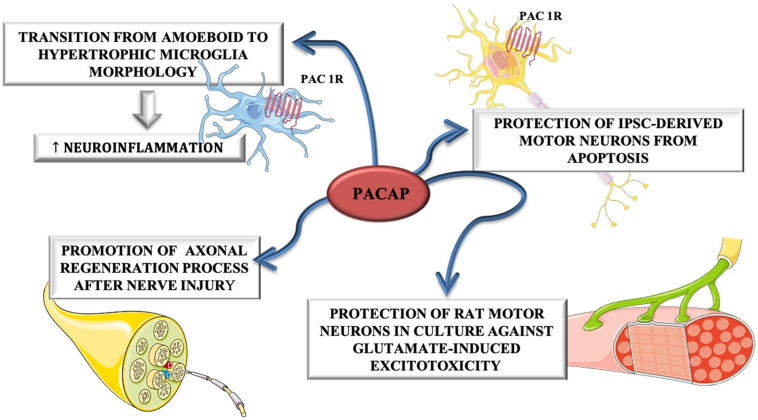
The dual role of PACAP on MNs and glial cells. PACAP induced neuroinflammation by promoting transition from amoeboid to hypertrophic microglia morphology in SOD1(G93A) mice. On the other hand, it promoted axonal regeneration process after nerve injury and protected rat motor neurons in culture against glutamate-induced excitotoxicity and iPSC-derived motor neurons from apoptosis.

Despite these evidences, Ringer et al. (2013), have highlighted that PACAP exerts a contradictory role during ALS progression. In fact, in the early phase of disease, it plays a neuroprotective role by promoting MNs survival, whereas in the end-stage of disease, PACAP promotes neuro-inflammation by stimulating microglial cells contributing to MNs degeneration. The dual role of PACAP on MNs and glial cells was depicted in Figure 4.

Apparently, conflicting data exist regarding PAC1R expression. Indeed, it is highly expressed in hypoglossal nucleus compared to oculomotor nucleus in healthy rat brains or after a moderate traumatic brain injury (Shioda et al., 1997; Skoglosa et al., 1999). However, the differential expression analysis of PACAP and PAC1R in oculomotor versus hypoglossal nucleus in ALS animal models or patients has not been performed, yet. To this regard, the expression of PAC1R could change in relation to MNs degeneration during different ALS-disease stages.

It has been hypothesized that PACAP, binding to PAC1R, activates the MAPK survival pathway directly or indirectly through EGFR trans-activation (Junier et al., 1993; Ayuso-Sacido et al., 2010; Moody et al., 2017; Maugeri et al., 2019a, b). In accord, in a meta-analysis study comparing microarray datasets of SALS motor cortex patients to mSOD1 G93A mice brains has been identified a commonly up-regulation of EGFR in both groups respect to controls (Morello et al., 2017b). Therefore, it has been speculated that the neuroprotective role played by PACAP in ALS may be direct or mediated by EGFR-phosphorylation. To this regard, it has been demonstrated that PACAP prevents epidermal growth factor (EGF) deprivation−induced cell death in NSC−34 cells expressing G93A SOD1 mutation, an *in vitro* model of ALS (Jiménez Garduño et al., 2017; Maugeri et al., 2019c). This neuroprotective effect is mediated by EGFR-phosphorylation leading to MAPK or PI3K signaling cascade activation. Moreover, PACAP modulated hypoxia-induced autophagy dysregulation through MAPK/ERK signaling activation in SOD1 G93A cells (D’Amico et al., 2020). Accordingly, high levels of ERK and AKT, two regulators of these pro-survival pathways, have been found in adult human oculomotor nucleus and may be in part responsible for the resilience of its MNs during ALS degeneration (Allodi et al., 2016).

The data shown in the present review present several limitations considering that most of the studies were performed in SOD1 G93A mice model at different disease phases (Mesnard et al., 2011; Ringer et al., 2013) and in brain samples of ALS decedents, representing the end-stage of the disease.

Although SOD1 G93A mice is the widely used animal model, reproducing motor defects similar to that observed in ALS-affected patients, there are important caveats to acknowledge. Firstly, the mSOD1 mouse model has a propensity to spontaneously delete copy number which can alter the severity of disease presentation (Zwiegers et al., 2014; Lutz, 2018); secondly, the overexpression of human wild-type SOD1 causes axonopathy in mice, challenging the role of the mutation as the driver of pathology (Joyce et al., 2011).

Moreover, many other genes are involved in sporadic forms of ALS in addition to SOD1 G93A mutation. Therefore, to better characterize the role of PACAP and its receptor on MNs survival in ALS, new studies should be performed in other transgenic animal models.

## Conclusion

**FIGURE 5:**
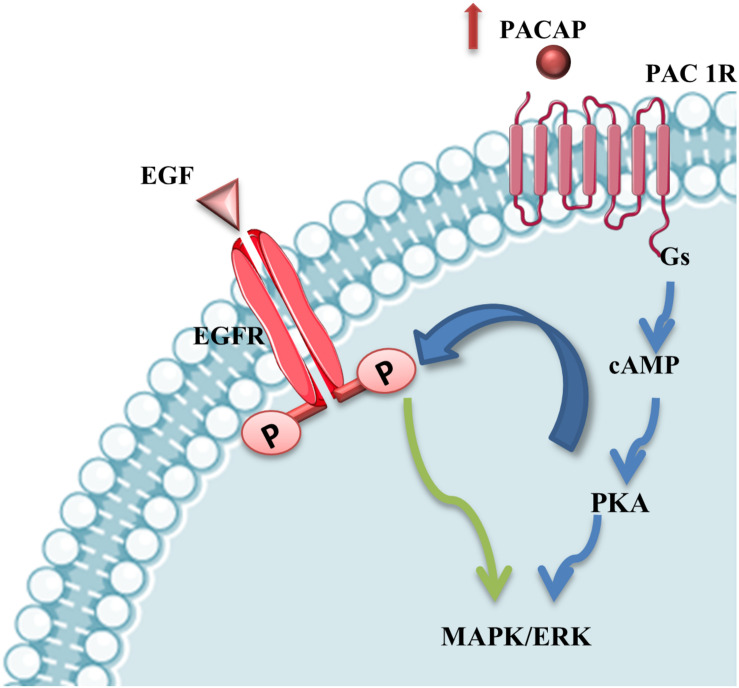
PACAP stimulation of MAPK/ERK survival pathway. PACAP binding to PAC1R activates MAPK/ERK survival pathway directly or through EGFR phosphorylation via PKA-signaling cascade stimulation in mSOD1 MNs.

The involvement of PACAP has been demonstrated in different neurodegenerative diseases, including MNs damage occurring in ALS. The evidences, above described, suggest that the different vulnerability of some cranial nerve motor nuclei could be also related to differential expression of PACAP in MNs. Moreover, PACAP’s protective effects in MNs during ALS progression could be direct through PAC1R activation or mediated by EGFR trans-activation promoting MAPK or PI3K signaling survival cascade (Figure 5).

However, too much is still unknown. To better characterize the pathogenetic mechanisms involved in this progressive neurodegenerative disease, it is advisable that the research community extends studies of gene expression and transcriptomes on samples from ALS decedents obtained at autopsy and from stereotactic biopsies on living patients when available.

## Author Contributions

GMa and VD’A designed the research projects and wrote the manuscript. AD’A, DR, and SC contributed to the manuscript preparation. All authors contributed to the article and approved the submitted version.

## Conflict of Interest

The authors declare that the research was conducted in the absence of any commercial or financial relationships that could be construed as a potential conflict of interest.
