# Supplementary material for: Differential Vulnerability of Oculomotor Versus Hypoglossal Nucleus During ALS: Involvement of PACAP
Source: Front Neurosci. 2020 Aug 11;14:805. doi: 10.3389/fnins.2020.00805 (PMC7432287; doi:10.3389/fnins.2020.00805)
Supplement: Supplementary file 1 [file Table_1.pdf]

Gene Symbol (Rattus)	CN3/4 versus CN12 and Cervical spinal cord (Hedlund et al 2010)	Gene Symbol (HUMAN)	SALS1 vs CTRL (Aronica et al 2015)	SALS2 vs CTRL (Aronica et al 2015)
Krt18	62.3	KRT18	1.8	2.9
Fkhr	48.5	FOXO1	1.5	-
**Gda**	**41.5**	GDA	-	-2.1
Cart	41.2	CARTPT	2.9	-
**Gda**	**39.7**	GDA	-	-2.1
Rttn	37.4	RTTN	-	-
Otx2	33.8	OTX2	-	-
Dpt	24.5	DPT	-	-4.1
Igsf1	23.7	IGSF1	-	-
Spint2	22.9	SPINT2	-	-4.4
Lamb1	21.4	LAMB1	2.6	-
Bcl11B	18.3	BCL11B	-4.2	-1.8
Irs4	17.7	IRS4	-	3.8
Lrit1	17.6	LRIT1	-	-
Ctsc	17.3	CTSC	-	-
RGD1306410 (Ccz1)	16.5	CCZ1	-	-
Rcn3	15.2	RCN3	-	2.8
Arpp-21	14.9	ARPP21	-	-
Cyp26B1	14.3	CYP26B1	-	-2.7
Enc1	12.7	ENC1	-	-5.1
Wbp5	12.6	WBP5	1.9	-
Synpr	12.3	SYNPR	-	-
Fchsd2	12.1	FCHSD2	-	-2.7
Irx2/Irx1	12.1	IRX1	-1.9	-
Necab1	12.0	NECAB1	-	-3.9
Coch	11.9	COCH	-	-
Syt1	11.6	SYT1	-	-3.7
RGD1559587	11.4	-	-	-
Pitx2	11.3	PITX2	-	-
Cplx3	11.0	CPLX3	-	-3.6
Cd63	10.4	CD63	2	-3
Foxp2	10.3	FOXP2	-	4.4
LOC68829 (Pde6g)	10.2	PDE6G	-	-
Slc13A4	10.2	SLC13A4	-	-
Irx1	9.8	IRX1	-1.9	-
Dhrsx	9.8	DHRSX	-	-
Ywhae/Doc2B	9.7	YWHAE	2.2	-3.1
**Gucy1A3**	**9.6**	GUCY1A3	-	-2.5

Ankrd28	9.5	ANKRD28	-	-2.2
Grp	9.4	GRP	-	-2.2
Adcyap1	9.3	ADCYAP1	-	-2.1
RGD1307215 (Arpp21)	9.1	ARPP21	-	-
Ssg1 (Ccdc80)	9.0	CCDC80	2	-
RGD131081 9 (Cracdl)	8.8	CRACDL	-	-
Rbp3	8.8	RBP3	-	-
Rreb1	8.6	RREB1	-3.5	-
Rgs16	8.4	RGS16	-	-2.2
Gad1	8.2	GAD1	-	-2.8
Etv1	8.2	ETV1	-	-
H2-T23	8.1	HLA-E	3.3	-2.6
Cd1D1	8.1	CD1D	-	-
Ifitm1	8.0	IFITM1	-	-3
Egr3/Bin3	8.0	EGR3/BIN3	-	-
Plac9	7.9	PLAC9	-	-
Opcml	7.8	OPCML	-	-3.9
Cpne7	7.7	CPNE7	-	-1.9
Anxa1	7.7	ANXA1	2.4	-
Kit	7.7	KIT	-	-
Ywhae/Doc2 B	7.7	YWHAE	2.2	-3.1
Col1A2	7.7	COL1A2	-	-
Oprm1	7.7	OPRM1	-	-
Xtrp3	7.6	SLC6A20	-2.7	-
Bcl11B	7.6	BCL11B	-4.2	-1.8
Slc6A7	7.6	SLC6A7	-	-
LOC498549 /RGD13087 72	7.6	AKAP11	-	-4.5
Cmya5	7.6	CMYA5	-	-3.1
Eef1A1	7.6	EEF1A1	3.1	-2.8
Lrrc38	7.5	LRRC38	-	5.6
G3Bp	7.4	G3BP1	2.2	-
Kcnf1	7.4	KCNF1	2.9	-
Cap2	7.3	CAP2	2.2	-
Rgs5	7.3	RGS5	-	-5.8
Dcn	7.2	DCN	-	-3.3
Tmem178	7.1	TMEM178A	-	-
Ptgds	7.1	PTGDS	-	-3
Slc39A4	7.0	SLC39A4	-	3.8
Cnr1	7.0	CNR1	-	-1.7
Ccnd2	7.0	CCND2	-	-4.1
Gch	6.7	GCH	-	4.6
Rasal1	6.7	RASAL1	-	-

LOC687064 (Col25a1)	6.7	COL25A1	-	2.4
Igf1	6.7	IGF1	-	-
Eps8L2	6.6	EPS8L2	-	2.3
Foxa2	6.6	FOXA2	-	-3.2
Fstl4	6.6	FSTL4	-	-
Myoz3/Synpo	6.5	SYNPO	-	-2.4
Eef1A1	6.5	EEF1A1	3.7	-3.2
Prrg2	6.5	PRRG2	-	1.9
Upp1	6.5	UPP1	-	-2.2
Fam132A	6.4	FAM132A	1.5	1.9
RGD1310251 (Mzb1)	6.4	MZB1	-	-
LOC690445	6.4	-	-	-
RGD1305038	6.4	-	-	-
Accn4	6.4	ASIC4	-	-
Calb1	6.3	CALB1	-	-
Gas7	6.3	GAS7	-	-2
Fscn2	6.3	FSCN2	-	-
Arhgef5	6.3	ARHGEF5	-	3.7
Dsc2	6.2	DSC2	-	-2.7
Bk	6.2	KNG1	-	-
**Egr1**	**5.2**	EGR1	-	-
**Igf2**	**2.5**	IGF2	-	-3.2
Hoxc5	383.3	HOXC5	-	-
Hoxd3/Hoxd1	78.9	HOXD1	-	-2.3
		HOXD3	-	-2.6
Hoxc8	47.1	HOXC8	-	3.7
Hoxc8	44.4	HOXC8	-	3.7
Crygn	42.9	CRYGN	-	3.3
Hoxd8/Hoxd4	39.3	HOXD8/HOXD	-	-
Hoxb5	37.8	HOXB5	-	-
Hoxa5	37.5	HOXA5	-	-
Hoxb4	26.6	HOXB4	-	-
RGD1561431 (Hoxa3)	15.8	HOXA3	-2	2
Hoxb2	15.3	HOXB2	-4	3.7
Calca	15.1	CALCB	-	-
Hoxa5	14.0	HOXA5	-	-
Hoxc4	11.9	HOXC4	-	-
Trim58/Olr1433	11.8	TRIM58	-2.8	-2.8
Fyxd7	11.5	-	-	-
Myh13	11.3	MYH13	-	6.1
Hoxd4	10.8	HOXD4	-	-

Glra1	10.3	GLRA1	-	-
Rspo3	10.1	RSPO3	-	-
Hspb1	9.7	HSPB1	2.2	-2.5
Bcl3	9.6	BCL3	1.8	-
Pcdhgb7	8.4	PCDHGB7	-	-
Trpv2	8.0	TRPV2	-	-1.8
Gabra2	7.6	GABRA2	-	-2.3
Pde4Dip	7.5	PDE4DIP	3.2	-2.23
Gbx2/RGD1559780	7.2	GBX2	-	26.75
Epha5	7.1	EPHA5	-	5.5
Nhlrc2/Adrb1	7.0	NHLRC2/ADRB1	-	-
Gabra2/Gabrg1	6.9	GABRG1	-	-
Tppp3	6.6	TPPP3	3.5	2.5
RGD1563303	6.6	-	-	-
Ldoc1	6.6	LDOC1	-	-
LOC360570 (Myo18a)	6.6	MYO18A	-	-
Pai2A	6.5	SERPINB2	-2.6	-
Angptl1/Rspo2	6.4	ANGPTL1	-	7.1
Meis2/LOC687952	6.4	MEIS2	1.7	2
Cd24	6.3	CD24	-	-1.6
Slc35d3	6.3	SLC35D3	-	-
LOC688954	6.3	-	-	-
RGD1562142	6.3	HOXB6	-	2.8
Clcn4-2	6.3	CLCN4	1.4	-
Trem2	6.3	TREM2	3.1	-
Bcl3	6.1	BCL3	1.8	-
Kcna1	6.1	KCNA1	-	-
RGD1563327	5.9	-	-	-
Hod	5.8	HOPX	1.9	-4.9
St6Gal1	5.7	ST6GAL1	1.9	-
Trhr	5.7	TRHR	-	-1.9
Htr2A	5.6	HTR2A	-	-2.8
RGD1306991	5.6	LAMP5	-	-
Sh3Bgrl3	5.6	SH3BGRL3	-	-2.7
Prph	5.5	PRPH	-	-2.3
Mtmr2	5.5	MTMR2	-	-2.6
Casc3	5.5	CASC3	-	-2
Chrm2/Ptn	5.5	CHRM2<br>PTN	-<br>-	4.27<br>-3
Frem3	5.4	FREM3	-	-

Scx	5.4	SCX	-	-
Wdr33	5.4	WDR33	1.7	-
Fbxo36	5.3	FBXO36	-	4.3
Clic3	5.3	CLIC3	-	4.4
Epha3	5.3	EPHA3	-	3.5
Atp2B3	5.3	ATP2B3	1.8	-3.3
RGD1563678	5.1	-	-	-
LOC682060/RGD1564733	5.1	-	-	-
Rab3B	5.1	RAB3B	-	-4.3
RGD1562378	5.1	H4C15; H4C1	-	-
A3Galt2/Zfp362	5.0	ZNF362	-	-2.5
Gas6	5.0	GAS6	-	2.08
RGD1562411/Rps6Kb1	5.0	RPS6KB1	1.5	-
Mocs3/Kcng1	5.0	IOCS3/KCNG	-	-3.1
Chrm2/Ptn	5.5	CHRM2	-	4.27
		PTN	-	-3
Tmem16C	4.9	ANO3	-	-2.2
Parva	4.9	PARVA	2	-
S100A10	4.9	S100A10	2.8	-
RGD1560587	4.9	EPHA4	-	-2.1
Itga7	4.8	ITGA7	2.2	-
RGD1565975/Irx3	4.8	IRX3	-1.6	3.6
Pmp22	4.8	PMP22	-	-3.4
Slc16A13	4.7	SLC16A13	-	-
Vipr2	4.7	VIPR2	-	-
Ogfrl1	4.7	OGFRL1	1.6	-
Megf11	4.7	MEGF11	-	-
Cacna1B	4.6	CACNA1B	-	-4.5
Pcdh17	4.6	PCDH17	-	-2.1
Ykt6	4.6	YKT6	2.5	-
Foxk2	4.6	FOXK2	2	-
Mmp28	4.5	MMP28	-	-2.9
Efna1/Efna3	4.5	EFNA1	1.7	-2.4
		EFNA3	-	2.9
Lphn3	4.5	LPHN3	-	-
Susd2	4.5	SUSD2	-	-2.4
Senp5	4.4	SENP5	-	-2.4
Lamp2/Atp1B4	4.4	LAMP2	-	-3.4
		ATP1B4	-	9.9

Sncg	4.4	SNCG	-	-
Fam159B	4.4	FAM159B	-	-
Ppm2C	4.4	PPM2C	-	-4.8
Stbd1	4.4	STBD1	-	8.14
Dysf	4.4	DYSF	-1.8	-
Bace2/Mx1	4.3	BACE2	-	-3.6
		MX1	-	-3.05
LOC679020	4.3	-	-	-
**Pgf**	**3.5**	PGF	-	-
